# Erratum: High performance platinum single atom electrocatalyst for oxygen reduction reaction

**DOI:** 10.1038/ncomms16160

**Published:** 2017-09-27

**Authors:** Jing Liu, Menggai Jiao, Lanlu Lu, Heather M. Barkholtz, Yuping Li, Ying Wang, Luhua Jiang, Zhijian Wu, Di-jia Liu, Lin Zhuang, Chao Ma, Jie Zeng, Bingsen Zhang, Dangsheng Su, Ping Song, Wei Xing, Weilin Xu, Ying Wang, Zheng Jiang, Gongquan Sun

Nature Communications 8: Article number: 15938 ; DOI: 10.1038/ncomms15938 (2017); Published: 07 24 2017; Updated: 09 27 2017.

The affiliation details for one of the corresponding authors, Ying Wang, are incorrect in this Article. This author is incorrectly affiliated with ‘University of Chinese Academy of Sciences, Beijing 100049, China.’ The correct affiliation details for this author are given below:

State Key Laboratory of Rare Earth Resource Utilization, Changchun Institute of Applied Chemistry, Chinese Academy of Sciences, Changchun 130022, China.

