# Supplementary material for: High performance platinum single atom electrocatalyst for oxygen reduction reaction
Source: Nat Commun. 2017 Jul 24;8:15938. doi: 10.1038/ncomms15938 (PMC5527280; doi:10.1038/ncomms15938)
Supplement: Supplementary Information [file ncomms15938-s1.pdf]

Type of file: PDF
Size of file: 0 KB
Title of file for HTML: Supplementary Information
Description: Supplementary Figures, Supplementary Tables, Supplementary Note and Supplementary References.

Type of file: PDF
Size of file: 0 KB
Title of file for HTML: Peer Review File
Description:

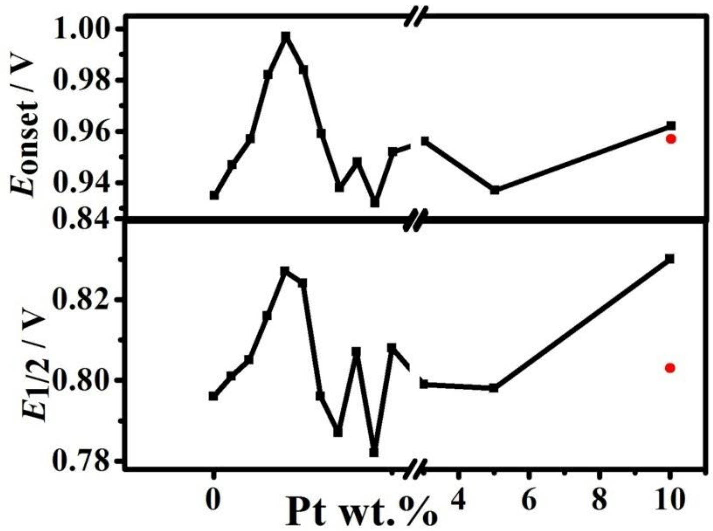

Supplementary Figure1. The optimization of Pt loading on Pt-N/C catalysts for ORR. All these catalysts are based on the fixed initial ratio of urea/carbon of 10 with fixed pyrolysis temperature of 950°C. The red point indicates the performance of pure Pt/C with Pt 10 wt%.

Supplementary Figure 1 shows clearly that the optimal Pt content is about 0.4 wt%.

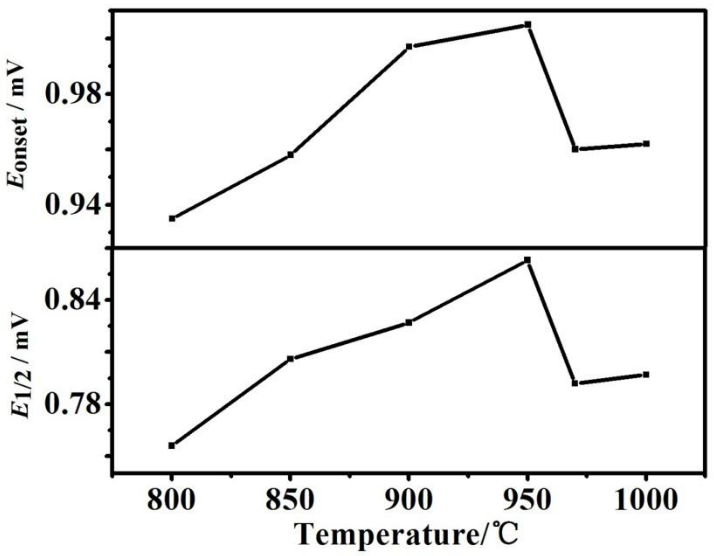

Supplementary Figure 2. The optimization of pyrolyzing temperature for Pt-N/C catalysts for ORR. All these catalysts are based on the fixed initial ratio of urea/carbon of 10 with fixed Pt content of 0.4wt% obtained from Supplementary Figure 1.

Supplementary Figure 2 shows clearly that the optimal pyrolyzing temperature for Pt-N/C is at 950°C.

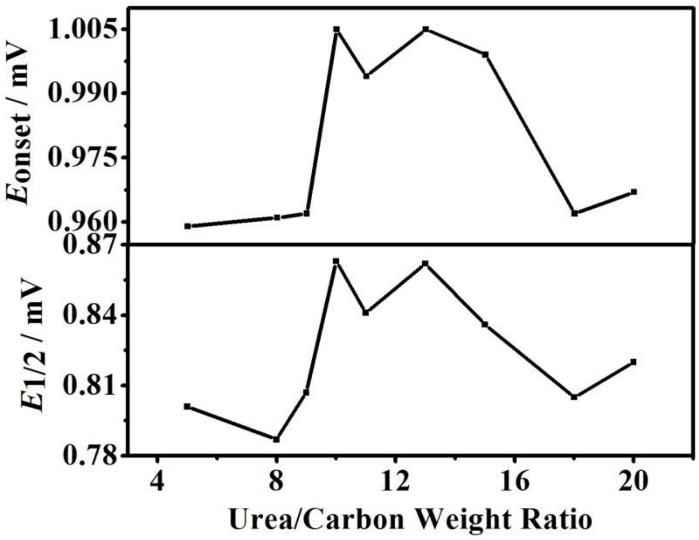

Supplementary Figure 3. The optimization of initial ratio of urea/carbon for Pt-N/C catalysts for ORR. All these catalysts are based on the optimized pyrolyzing temperature of 950°C (obtained from Supplementary Figure 2) and fixed Pt content of 0.4wt% obtained from Supplementary Figure 1.

Supplementary Figure 3 shows clearly that the optimal initial weight ratio of urea/carbon for Pt-N/C catalysts is 10.

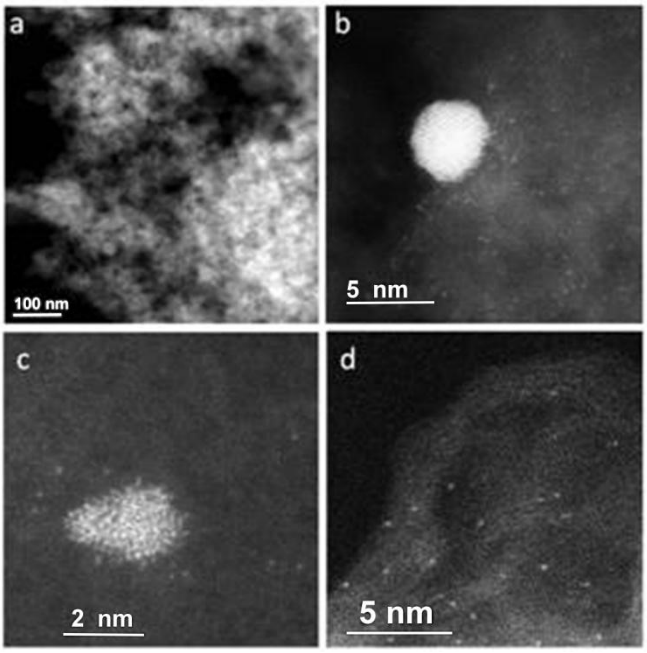

Supplementary Figure 4. (a)Low-magnified HAADF-STEM image of Pt_1-N/BP.(b, c) More HAADF-STEM images of Pt_1/BP to show the coexistence of individual Pt single atoms with small Pt nanoparticles. (d)HAADF-STEM images of Pt_1-N/BP after 18k CV cycles in O_2-saturated 0.1 M KOH. Statistically, the surface density of Pt atoms didn't show obvious decrease after 18k CV cycles in O_2-saturated 0.1 M KOH, consistent with the high long-term stability of ORR activity observed on electrode (Figure 2f and Supplementary Figures 10c and10d).

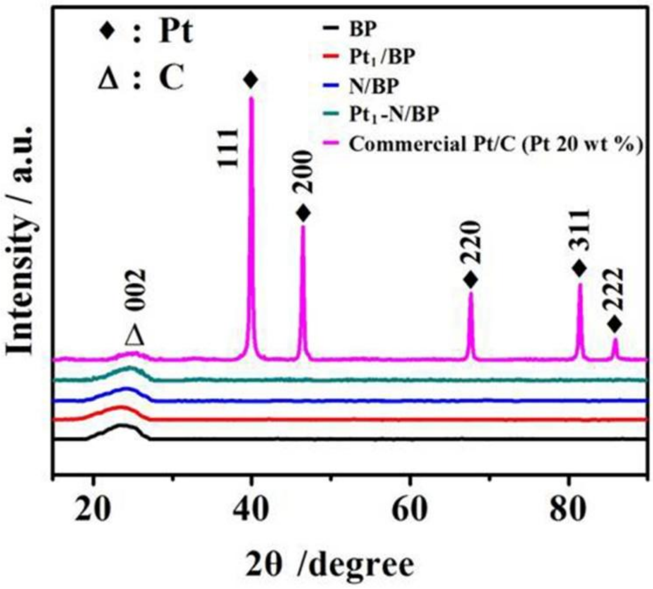

Supplementary Figure **5** X-ray diffraction (XRD) patterns of different samples: BP, Pt_1/BP, N/BP, Pt_1-N/BP. For comparison, the result for commercial Pt/C was also presented in red curve.

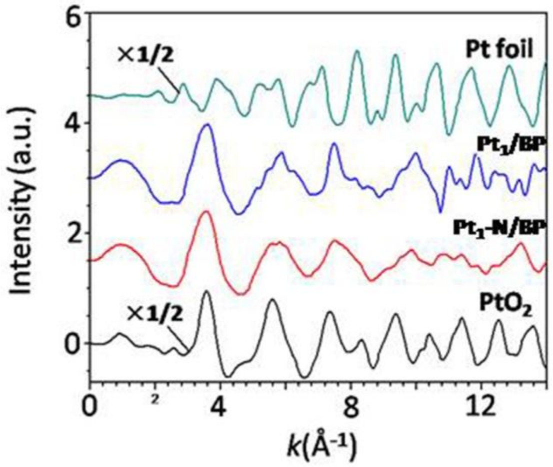

Supplementary Figure 6. Pt L3-edge EXAFS oscillation function $k^2 \cdot \chi(k)$ on different substrates: Pt foil, Pt_1/BP, Pt_1-N/BP, PtO_2. Pt L3-edge XAFS measurements were performed at Beam line 14W of the SSRF. The storage ring was operated at 3.5 GeV with a maximum current of 240 mA. The incident X-ray was monochromatized by using a doublecrystal Si (111) monochromator. The data were collected in fluorescence and transmission modes for the target and reference samples, respectively. The XAFS data were analyzed by using the Demeter software package[1] (University of Chicago).The theoretical scattering amplitude and phase-shift functions of all the paths for fitting the EXAFS data were calculated by FEFF6 code (University of Washington)[2-4].

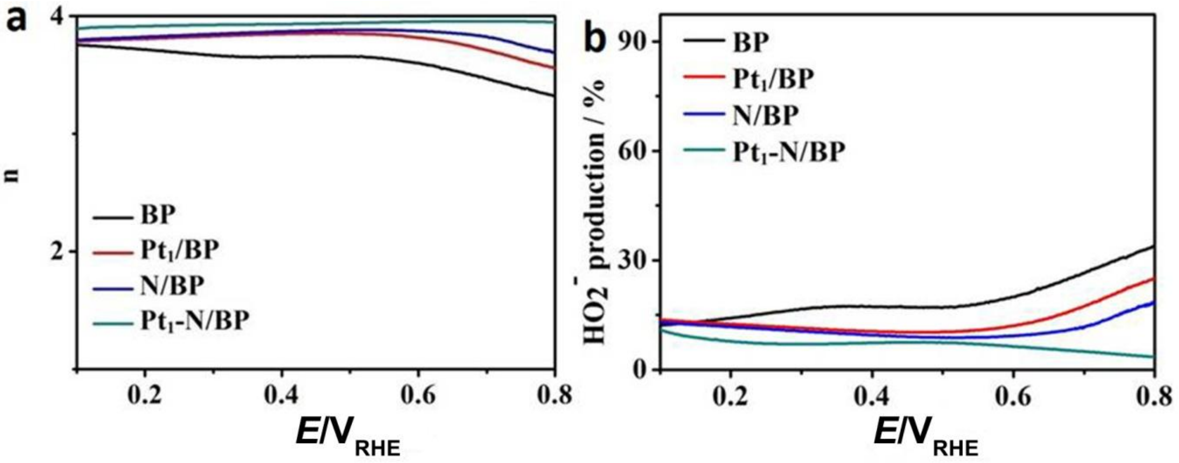

Supplementary Figure 7. (a)The electron transfer number (n) and the H_2O_2 yield (b) on different catalysts of BP, Pt_1/BP, N/ BP and Pt_1-N/BP in 0.1 M $HClO_4$.

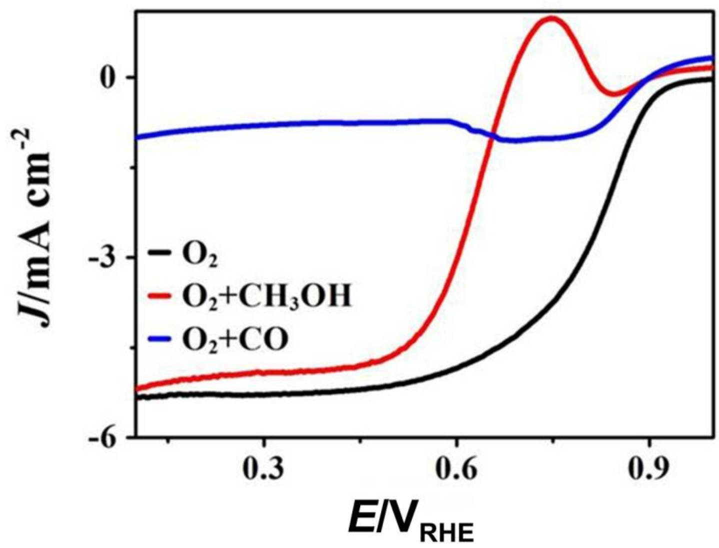

Supplementary Figure 8. The response of commercial Pt/C to O_2, CO and methanol (0.5 M) in 0.1 M $HClO_4$.

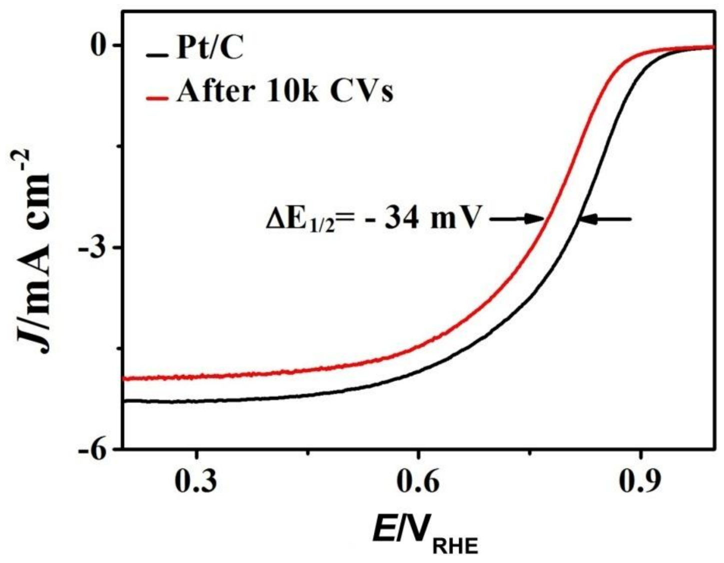

Supplementary Figure 9. The long-term operation stability of commercial Pt/C with Pt loading of 20 wt.% in0.1 M $HClO_4$.

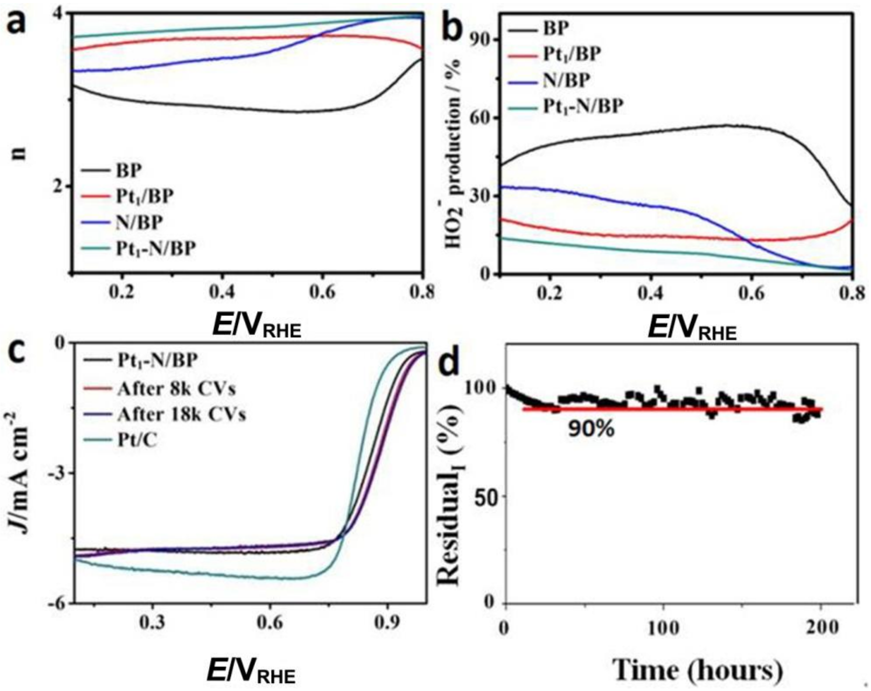

Supplementary Figure 10. (a) The electron transfer number (n) and the H_2O_2 yield (b) on Pt_1-N/BP in 0. 1 M KOH.(c) The durability study of Pt_1-N/BP in O_2-saturated 0.1 M KOH. (d)The acidic fuel cell lifetime with Pt_1-N/BP as cathode ($Residual_I$: the remains of current) test at fixed potential of 0.5 V and 70 ℃ for 200 hours.

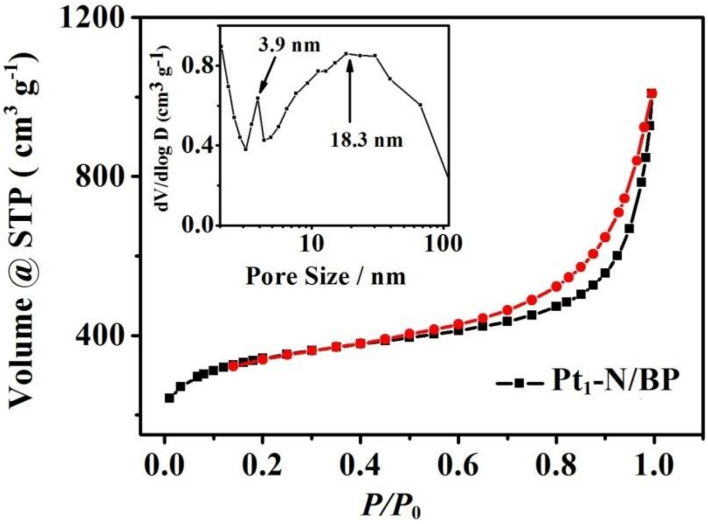

Supplementary Figure 11. N_2 sorption isotherms of Pt_1-N/BP. Inset: the pore size distribution.

Supplementary Table 1. Comparison of $E_{1/2}$ between Pt_1-N/BP and some other typical best non-Pt ORR electrocatalysts in acidic condition

Samples	$E_{1/2}$(vs.RHE)	References
Pt_1-N/BP	0.76 V(0.1 M $HClO_4$)	In this work
Fe_3C/NG-800	0.77 V(0.1 M $HClO_4$)	Ref. 5
Fe_3C/C-700	0.73 V(0.1 M $HClO_4$)	Ref. 6
Fe–N/C-800	0.62 V(0.1 M $HClO_4$)	Ref. 7
Fe_2 N/N-GAs-20	0.71 V (1.0 M $HClO_4$)	Ref. 8
Fe-N-CNFs	0.61V (0.5 M H_2SO_4)	Ref. 9
Fe/N/CF	0.80 V (0.5 M H_2SO_4)	Ref. 10
PANI-Fe-C	0.80 V (0.5 M H_2SO_4)	Ref.11

Supplementary Table 2. Typical best non-Pt ORR electrocatalysts in alkaline condition (0.1 M KOH)

Samples	$E_{1/2}$(0.1 M KOH)(vs.RHE)	References
Pt_1-N/BP	0.87 V	In this work
{Co}-[FeCo]O_4/NG	0.86 V	Ref.12
Co,N-CNF	0.82 V	Ref. 13
PdCuCo NCs/C	0.87 V	Ref. 14
NPMC-1000	0.85 V	Ref.15
Fe-N-CNFs	0.83 V	Ref.9
Fe-N/C-800	0.82 V	Ref. 16
Fe–N/C-800	0.81 V	Ref.7
N-Fe-CNT/CNP	0.87 V	Ref.17
VA-NCNT/GC	0.86 V	Ref 18.
Co SAs/N-C(900)	0.88V	Ref.19

Supplementary Table 3. Acidic H_2-O_2 Fuel cell performance and Pt consumption in whole cell at peak power density and 80°C

Samples	Pt consumption/ g_{Pt} kW^{-1}	References
**Pt_1-N/BP**	**0.13 g_{Pt} kW^{-1}**	**In this work**
Fe/N/C	0.33 g_{Pt} kW^{-1}	Ref.20
S-Fe/N/C	0.39 g_{Pt} kW^{-1}	Ref.21
Fe/N/C	0.33 g_{Pt} kW^{-1}	Ref.10
PANI-Fe-C	0.45 g_{Pt} kW^{-1}	Ref.11

Supplementary Table 4. Durability tests of catalysts in acidic H_2/O_2 fuel cell at fixed potential of 0.5 V and comparison of the durability between our Pt single atom catalyst and some other Pt-free ORR catalysts in fuel cells at fixed potential of 0.5 V. In all these cases, the anodes of the fuel cells were all based on Pt/C for hydrogen oxidation reaction.

Samples	Current remained after a period of time at 0.5 V	References
**Pt_1-N/BP**	**74% remained after 200 hours at 80°C**	**In this work**
**Pt_1-N/BP**	**90% remained after 200 hours at 70°C**	**In this work**
S-Fe/N/C	28% remained after 100 hours at 80°C	Ref. 21
Fe/N/C	44% remained after 100 hours at 80°C	Ref.22

Supplementary Note 1: Computational details

All the electronic structure calculations have been carried out through the spin-polarized density functional theory (DFT) calculations as implemented in the Vienna ab initio simulation package (VASP)[23-26].Projector augmented wave (PAW) based potentials were used to describe the interactions between valence electrons and ion cores[27,28]. The Perdew−Burke−Ernzerhof form of the Generalized-Gradient Approximation was employed to describe electron exchange and correlation[29]. The wave functions at each k-point were expanded with a plane wave basis set and a kinetic cutoff energy up to 400 eV. Brillouin zone integration was approximated by a sum over special selected k-points using the Monkhorst–Pack method[30] and they were set to 5×5×1. The electron occupancies were determined using Gaussian broadening with a width of 0.05 eV. Geometries were optimized until the energy was converged to 1.0×10^{-5}eV per atom and the force was converged to 0.01 eVÅ^{-1}. A supercell of 7.38×7.38 Å in sized was used with a vacuum space of 25 Å for graphene-based structures. For Pt (111), a 3×3supercell and three-layer slab is utilized. The bottom layer is fixed to mimic Pt bulk.

The formation energy (E_f) was calculated as follows:

$$E_f = E_{total} - \sum_x n_x \mu_x \quad (3)$$

where E_{total} is the total energy of the whole system, n_x is the number of C, N and Pt atoms and the corresponding chemical potentials were derived from graphene, gaseous N_2 and a Pt atom in vacuum, respectively.

The adsorption energy (E_{ads}) of an adsorbate was defined as follows:

$$E_{ads} = E_{substrate+adsorbate} - E_{adsorbate} - E_{substrate} \quad (4)$$

where $E_{substrate+adsorbate}$, $E_{adsorbate}$, and $E_{substrate}$ stand for the total energies of a substrate and adsorbate, a gas phase adsorbate, and an isolated substrate, respectively. According to such a definition, a more negative value of E_{ads} signifies greater thermodynamic stability of the composite system.

Based on our experimental observations (Supplementary Figure 7, 10), the four-electron ORR mechanisms were investigated on the various graphene-based structures (Supplementary Figure12, 13) in the present work. In alkaline medium each ORR step can be summarized as follows,

$O_2 + 2H_2O + 4e^- \longrightarrow {}^*O_2 + 2H_2O + 4e^-$ (5)

${}^*O_2 + 2H_2O + 4e^- \longrightarrow {}^*OOH + OH^- + H_2O + 3e^-$ (6)

${}^*OOH + OH^- + H_2O + 3e^- \rightarrow 2OH^- + {}^*O + H_2O + 2e^-$ (7)

$2OH^- + {}^*O + H_2O + 2e^- \rightarrow 3OH^- + {}^*OH + e^-$ (8)

$3OH^- + {}^*OH + e^- \rightarrow 4OH^-$ (9)

Overall : $O_2 + 2H_2O + 4e^- \rightarrow 4OH^-$ (10)

The free energy diagrams of the oxygen reduction reactions (ORR) have been calculated according to the method developed by Nørskov *et al.*[31] Free energy change from initial states to final states of the reaction is calculated as follows:

$$\Delta G = \Delta E + \Delta ZPE - T\Delta S + \Delta G_U + \Delta G_{pH} + \Delta G_{field} \quad (11)$$

where ΔE is the total energy change obtained from DFT calculations, ΔZPE is the change in zero-point energy, T is the temperature (298.15 K), and ΔS is the change in entropy. $\Delta G_U = eU$, where U is the electrode potential with respect to standard hydrogen electrode, and e is the charge transferred. $\Delta G_{pH} = k_B T \ln 10 \times pH$, where k_B is the Boltzmann constant, and pH=0 for acid medium and 13 for alkaline medium in this study[32,33]. ΔG_{field} is the free energy correction due to the electrochemical double layer and is neglected as in previous studies[31,33]. Gas-phase H_2O at 0.035 bar was used as the reference state, because at this pressure gas-phase H_2O is in equilibrium with liquid water at 298.15 K. The free energy of O_2 was obtained from the free energy change of the reaction $O_2 + 2H_2 \rightarrow 2H_2O$, which is -4.92 eV at 298.15 K and a pressure of 0.035 bar[31]. The free energy of (H^++e^-) in solution at standard conditions of U=0 and pH=0 is equal to that of 1/2 H_2 according to a computational hydrogen electrode model suggested by Nørskov *et al.*[31], whereas at finite potential and pH=0, it is shifted by –eU, as used in some previous studies[31-34]. The free energy of OH^- was calculated from the reaction $H^+ + OH^- \rightarrow H_2O$, which is in equilibrium in water solution[32]. The entropies and vibrational frequencies of the species in gas phase were taken from the NIST database[35]. Zero-point energy and entropies of the adsorbed species were calculated from the vibrational frequencies. The DFT total energies, ZPE, TS, and Gibbs free energies of gas molecules and the ORR intermediates were listed in Supplementary Table 6, Supplementary Table 7, and Supplementary Table 8, respectively. Based on those data, we can calculate the free energy change ΔG of each step for the ORR on catalytic surfaces.

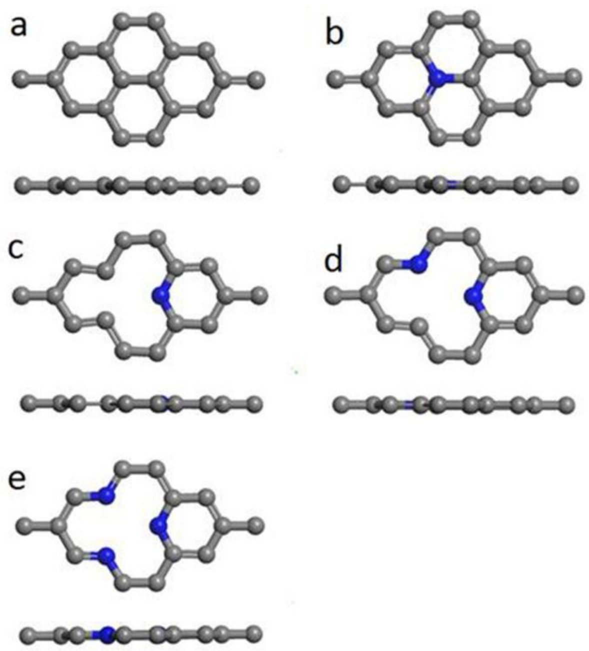

Supplementary Figure 12. Optimized structures of variations of graphene substrates. (a) pristine graphene, (b) graphitic N doped graphene (g-N), (c) pyridinic N1 doped graphene (g-P-N1), (d) pyridinic N2 doped graphene (g-P-N2), (e) pyridinic N3 doped graphene (g-P-N3). The grey and blue denote the carbon and nitrogen atoms, respectively.

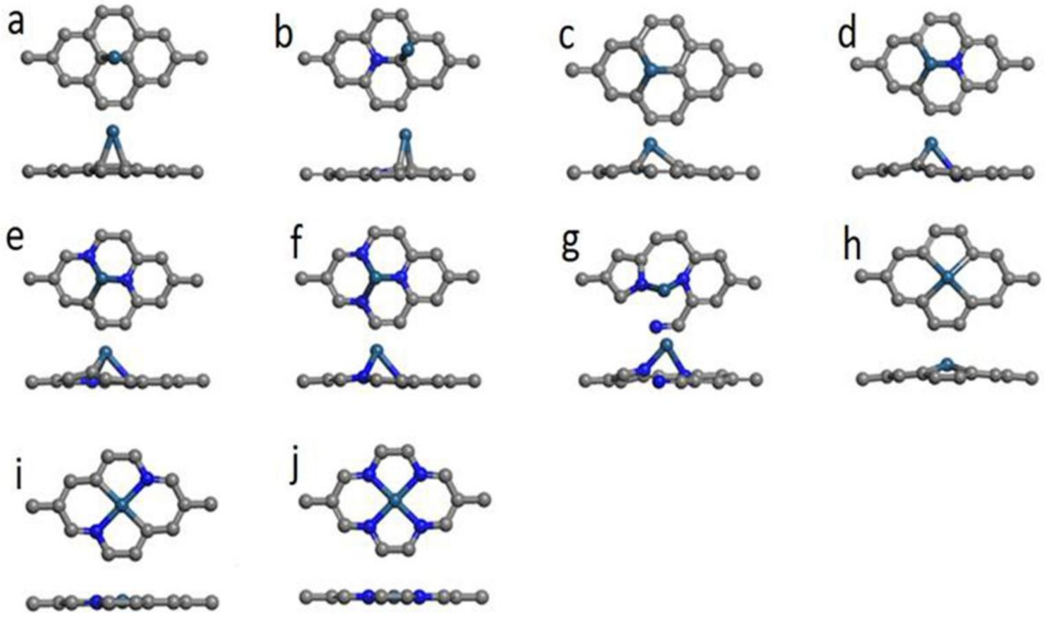

Supplementary Figure 13. Optimized structures Pt adsorbed on variations of graphene substrates. (a) g-Pt1, (b) g-N-Pt1, (c) g-1-Pt1, (d) g-P-N1-Pt1, (e) g-P-N2-Pt1, (f) g-P-N3-Pt1, (g) g-Py-N1-P-N2-Pt1, (h) g-2-Pt1, (i) g-2-P-N2-Pt1, (j) g-2-P-N4-Pt1. The grey, blue, and cyan balls denote the carbon, nitrogen, and platinum atoms, respectively.

Supplementary Table 5. The formation energy (E_f) of several graphene-based structures

structures	E_f(eV)
graphene	0
g-N	0.98
g-1	7.81
g-P-N1	6.12
g-P-N2	4.94
g-P-N3	3.56
g-2	8.82
g-2-P-N2	7.02
g-2-P-N4	3.76
g-Pt1	-1.56
g-1-Pt1	0.87
g-2-Pt1	1.18
g-N-Pt1	-1.05
g-P-N1-Pt1	0.78
g-P-N2-Pt1	0.52
g-P-N3-Pt1	0.63
g-2-P-N2-Pt1	-1.20
g-2-P-N4-Pt1	-3.50

As for the Pt1-based active site on Pt_1/BP, from the formation energies for the most possible/relevant sites (the part shown in Supplementary Table 5 in gray) on Pt_1/BP, one can tell that the formation of g-Pt1 (Supplementary Figure 13) site is much easier than then other two sites (g-1-Pt1 and g-2-Pt1); moreover, due to the extremely low surface defect site density on oxidized carbon (<5%)[36], one can tell that only g-Pt1 (Supplementary Figure 13) site could be the dominant active site on Pt_1/BP. This conclusion was further confirmed by the calculation which shows the active site (g-Pt1) tends to be oxidized by oxygen in air to a low active state for ORR (Figure 2 and Supplementary Figure 26-28) and also by the XPS analysis which shows the majority of Pt on Pt_1/BP is indeed oxidized (Figure 1d,e). In all, we can tell that the "real" main Pt1-based active site on Pt_1/BP is the oxidized g-Pt1 which possesses low ORR activity.

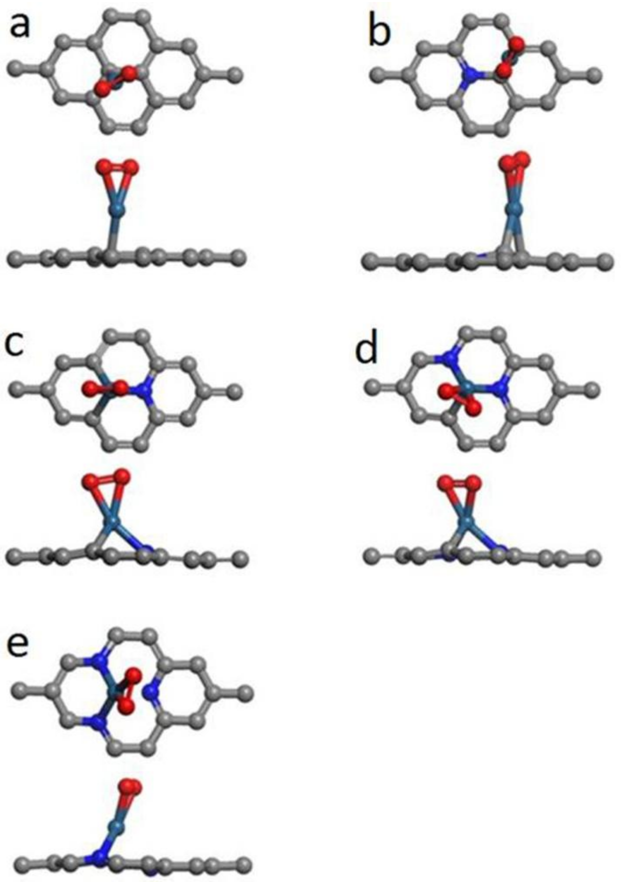

Supplementary Figure 14. Optimized configurations of O_2 adsorption on (a) g-Pt1, (b) g-N-Pt1, (c) g-P-N1-Pt1, (d) g-P-N2-Pt1, (e) g-P-N3-Pt1. The grey, blue, cyan and red balls denote the carbon, nitrogen, platinum and oxygen atoms, respectively.

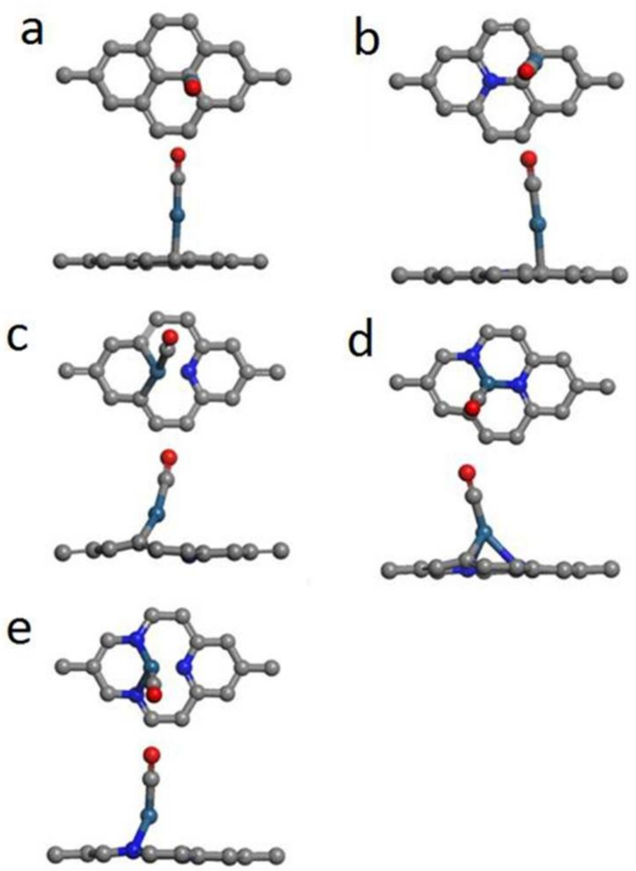

Supplementary Figure 15. Optimized configurations of CO adsorption on (a) g-Pt1, (b) g-N-Pt1, (c) g-P-N1-Pt1, (d) g-P-N2-Pt1, (e) g-P-N3-Pt1. The grey, blue, cyan and red balls denote the carbon, nitrogen, platinum and oxygen atoms, respectively.

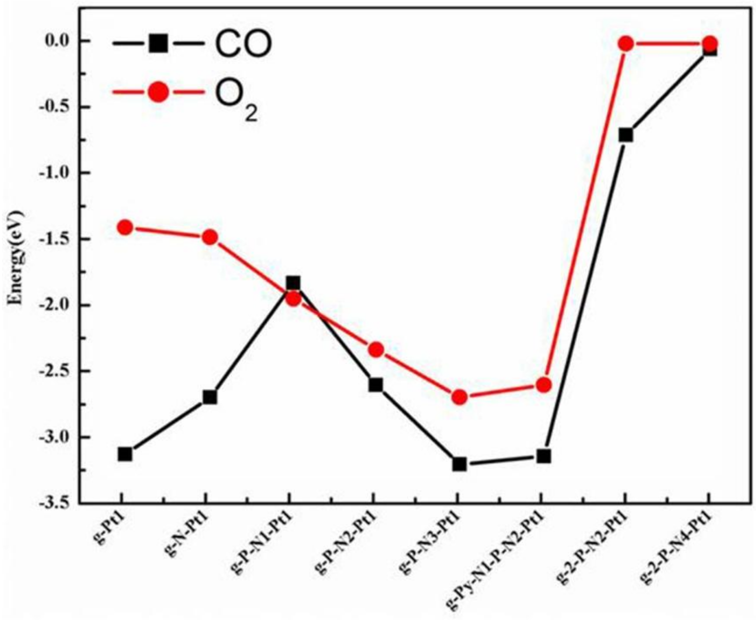

Supplementary Figure 16. The adsorption energy (E_{ads}) of O_2 and CO adsorbed on different substrates.

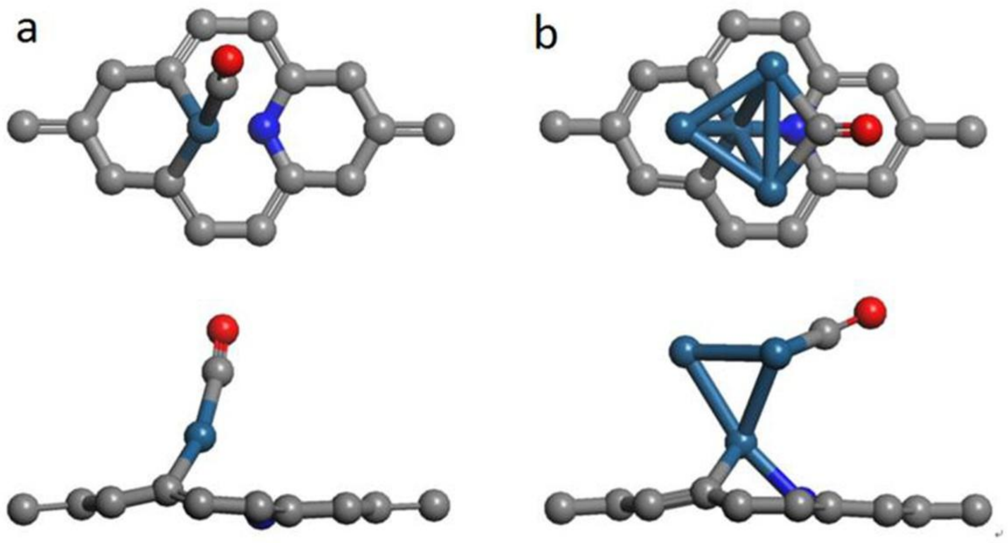

Supplementary Figure 17. Optimized configurations of CO adsorption on (a) g-P-N1-Pt1 and (b) g-P-N1-Pt4. The grey, blue, cyan and red balls denote the carbon, nitrogen, platinum and oxygen atoms, respectively.

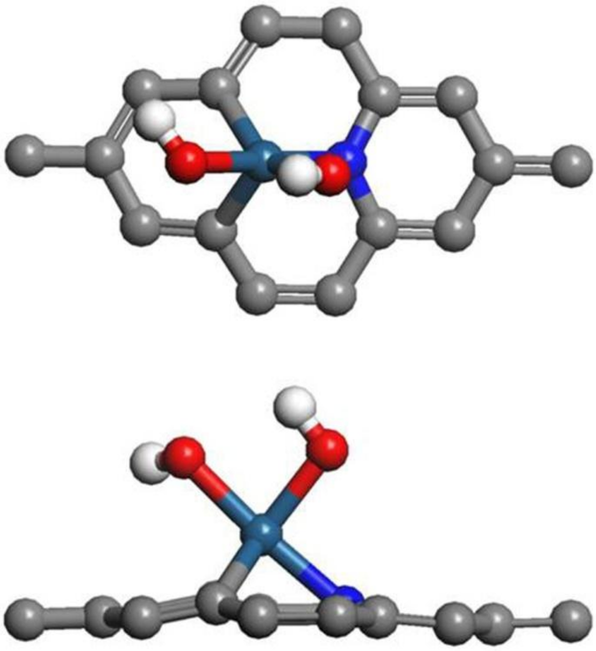

Supplementary Figure 18. Atomic structure of the lowest-energy configuration of decomposed H_2O_2 molecule adsorbed on g-P-N1-Pt1 substrate. The grey, blue, cyan and red balls denote the carbon, nitrogen, platinum and oxygen atoms, respectively.

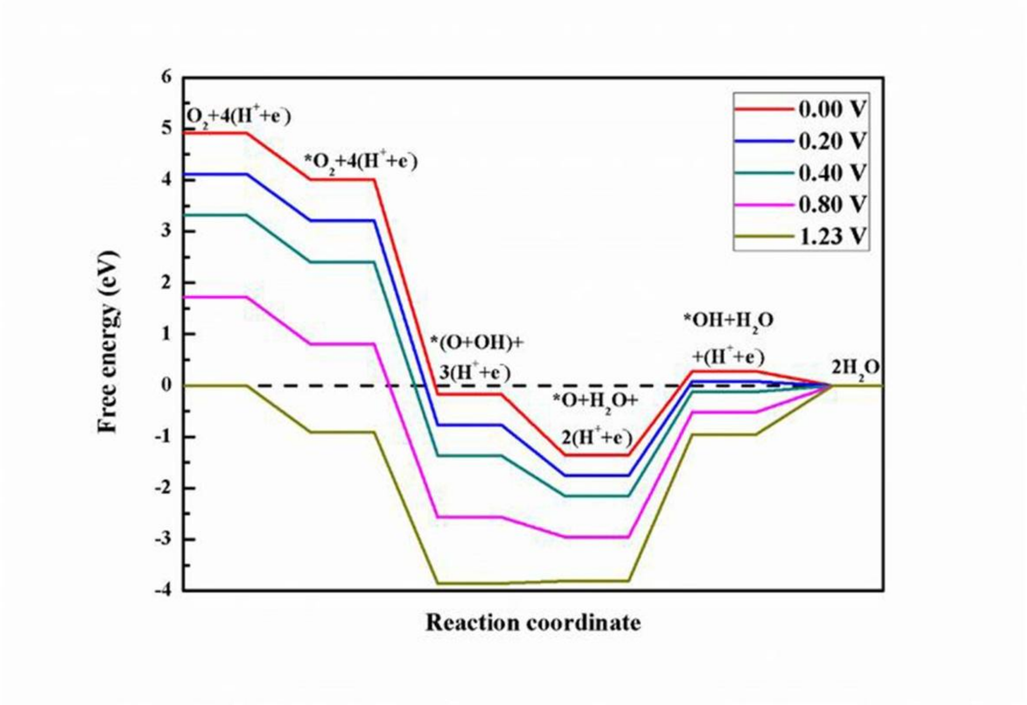

Supplementary Figure 19. Free energy diagram for complete O_2 reduction on the g-P-N1 substrate in acidic medium.

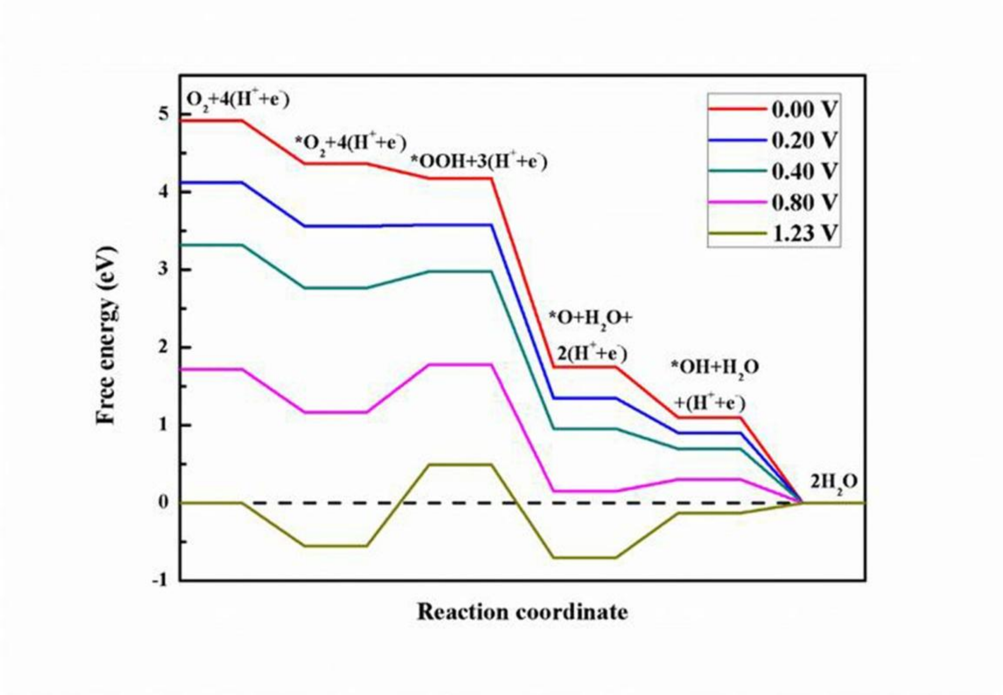

Supplementary Figure 20. Free energy diagram for complete O_2 reduction on the Pt (111) substrate in acidic medium.

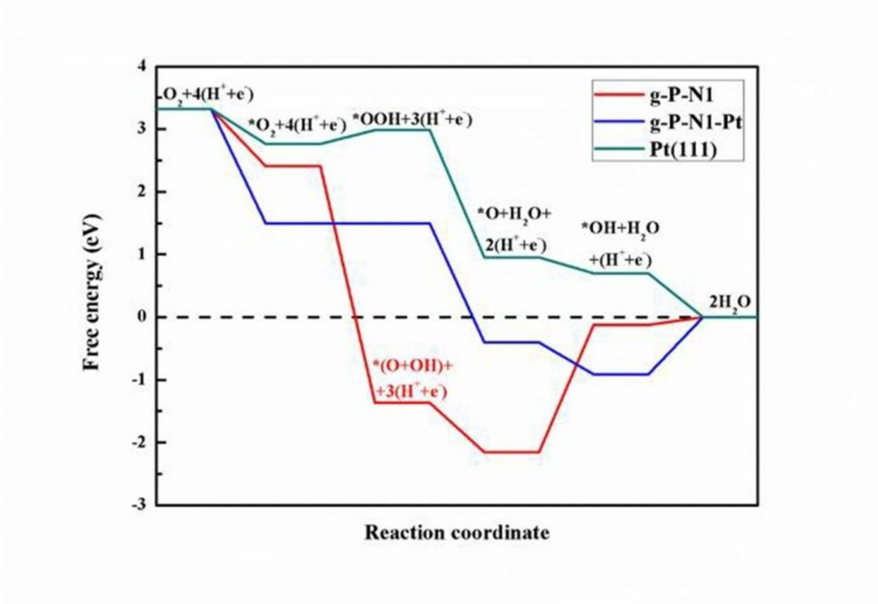

Supplementary Figure 21. Free energy diagram for complete O_2 reduction on the g-P-N1, g-P-N1-Pt1, and Pt (111) substrate in acidic medium at 0.40 V.

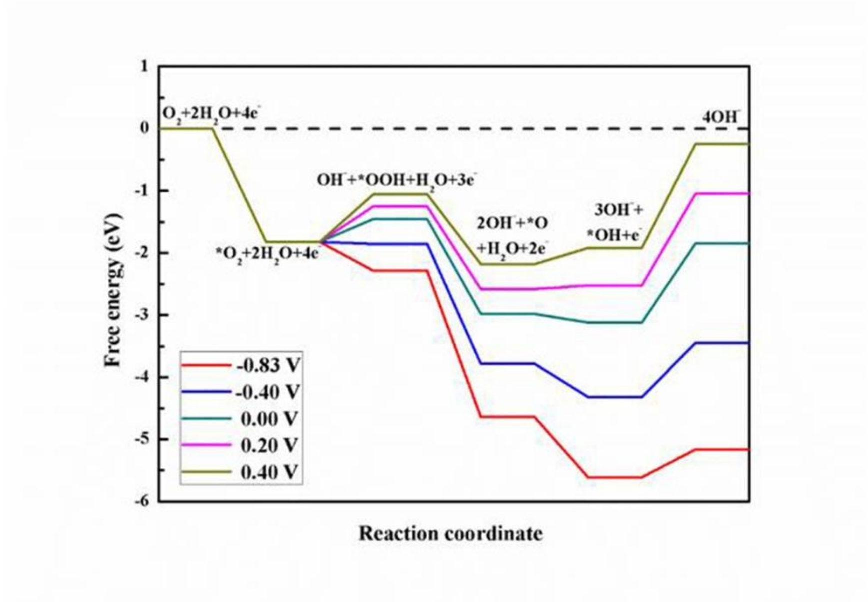

Supplementary Figure 22. Free energy diagram for complete O_2 reduction on the g-P-N1-Pt1substrate in alkaline medium.

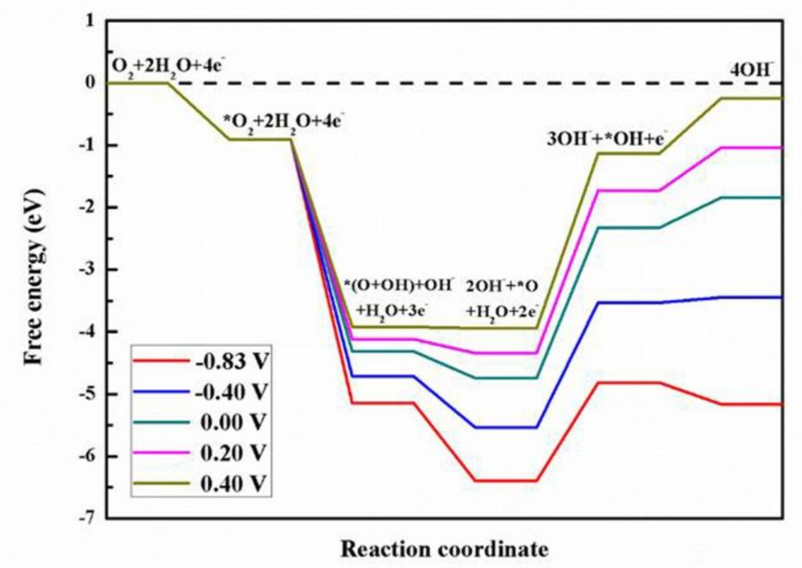

Supplementary Figure 23. Free energy diagram for complete O_2 reduction on the g-P-N1 substrate in alkaline medium.

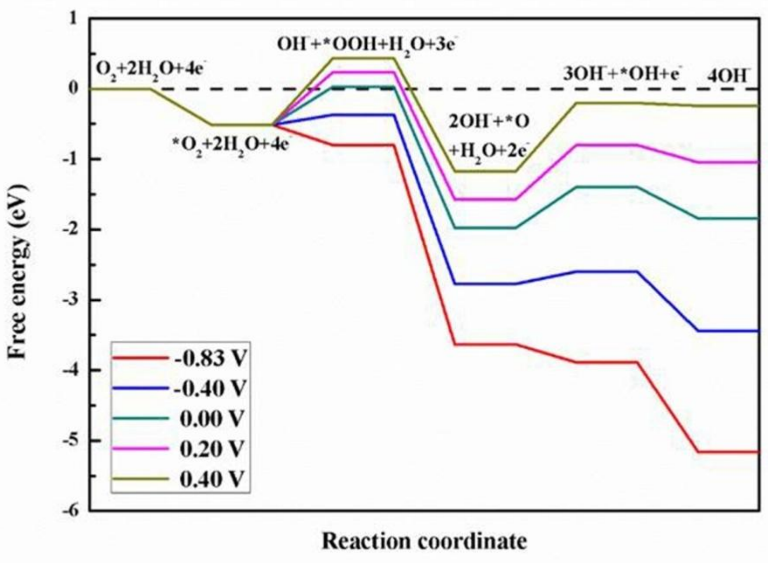

Supplementary Figure 24. Free energy diagram for complete O_2 reduction on the Pt(111)substrate in alkaline medium.

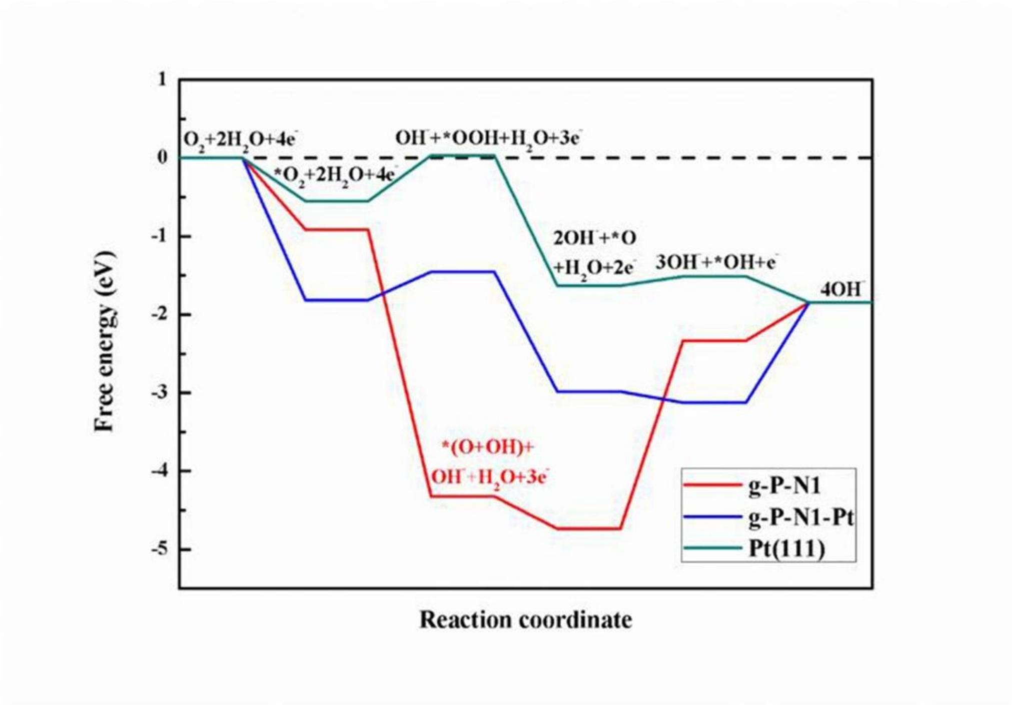

Supplementary Figure 25. Free energy diagram for complete O_2reduction on the g-P-N1, g-P-N1-Pt1, and Pt (111) substrate in alkaline medium at 0 V.

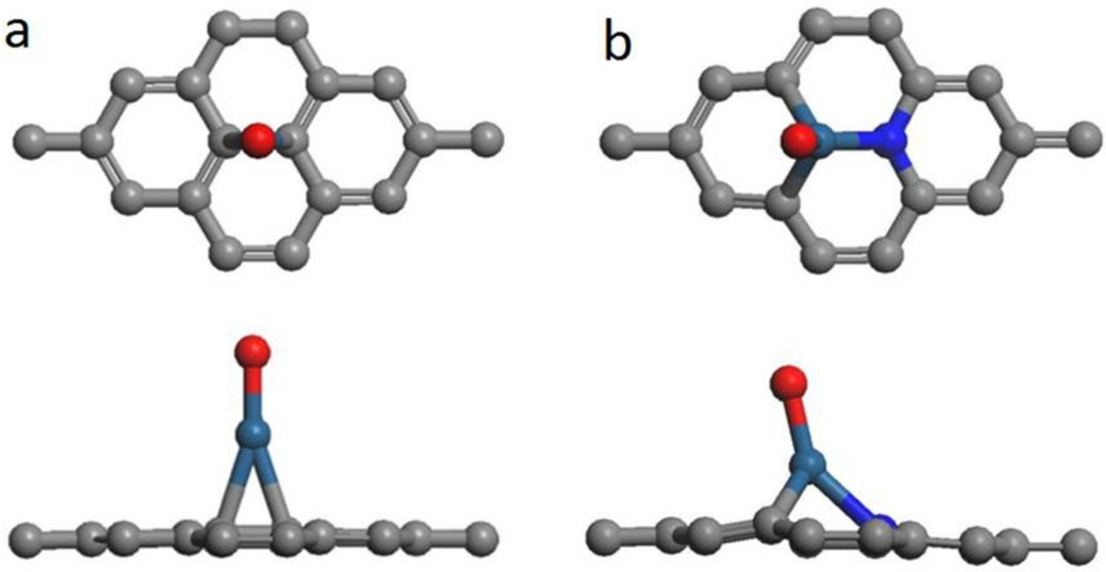

Supplementary Figure 26. Optimized configurations of oxidized g-Pt1 (a) and oxidized g-P-N1-Pt1 (b).

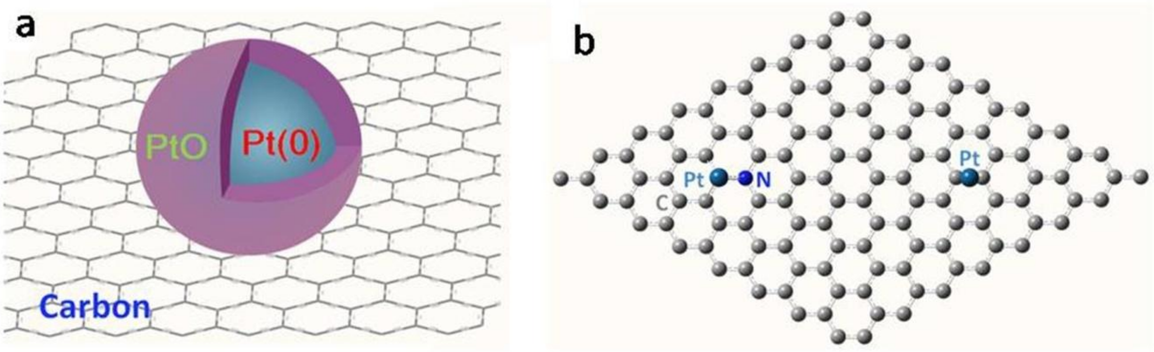

Supplementary Figure 27. (a) Scheme of a carbon-supported Pt nanoparticle with Pt(0) protected by the skin of Pt oxide; (b) two types of Pt1-based active sites on carbon: Pt_1-N_1/C and Pt_1/C.

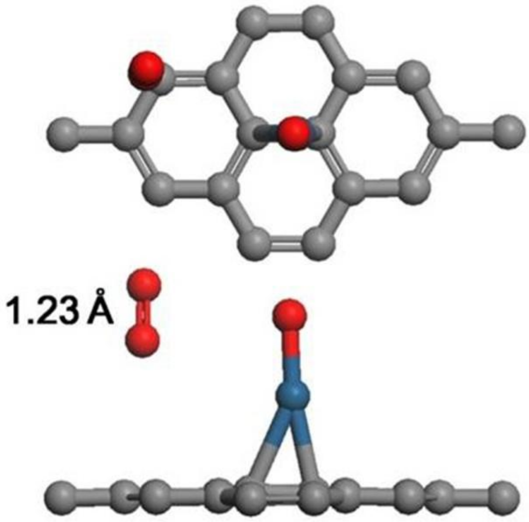

Supplementary Figure 28. Optimized configurations of O_2 adsorption on oxidized g-Pt1 site.

Supplementary Table 6. DFT total energies (E), zero-point energies (ZPE), entropies multiplied by T (= 298.15 K) (TS), free energies (G) of gas molecules and the total energies of catalytic surfaces.

	**E (eV)**	**ZPE (eV)**	**TS (eV)**	**G (eV)**
**H_2O**	**-14.236**	**0.559**	**0.670**	**-14.347**
**H_2**	**-6.760**	**0.273**	**0.404**	**-6.891**
**O_2**	**-9.858**	**0.098**	**0.634**	**-9.993**
**g-P-N1**	**-149.824**	**0**	**0**	**-149.824**
**g-P-N1-Pt1**	**-155.694**	**0**	**0**	**-155.694**
**Pt(111)**	**-152.734**	**0**	**0**	**-152.734**

Supplementary Table 7. DFT total energies (E), zero-point energies (ZPE), entropies multiplied by T (= 298.15 K) (TS), free energies (G) and relative free energies (ΔG) of ORR intermediates in acidic medium.

	E(eV)	ZPE(eV)	TS(eV)	G(eV)	ΔG_U (eV)	ΔG_{pH}(eV)	ΔG(eV)
**g-P-N1**							
**U=0.00 V**							
Step (1) *	-149.824	0.000	0.000	-149.824	0.00	0	4.92
Step(2) O_2*	-160.801	0.169	0.097	-160.729	0	0	4.008
Step(3)(O+OH)*	-168.774	0.511	0.087	-168.350	0.00	0	-0.168
Step(4) O*	-158.734	0.117	0.020	-158.636	0.00	0	-1.356
Step(5) OH*	-160.844	0.430	0.030	-160.445	0.00	0	0.281
**U=0.20 V**							
Step (1) *	-149.824	0.000	0.000	-149.824	0.20	0	4.12
Step (2) O_2*	-160.801	0.169	0.097	-160.729	0	0	3.208
Step(3)(O+OH)*	-168.774	0.511	0.087	-168.350	0.20	0	-0.768
Step(4) O*	-158.734	0.117	0.020	-158.636	0.20	0	-1.756
Step(5) OH*	-160.844	0.430	0.030	-160.445	0.20	0	0.081
**U=0.40 V**							
Step (1) *	-149.824	0.000	0.000	-149.824	0.40	0	3.32
Step (2) O_2*	-160.801	0.169	0.097	-160.729	0	0	2.408
Step(3)(O+OH)*	-168.774	0.511	0.087	-168.350	0.40	0	-1.368
Step(4) O*	-158.734	0.117	0.020	-158.636	0.40	0	-2.156
Step(5) OH*	-160.844	0.430	0.030	-160.445	0.40	0	-0.119
**U= 0.80 V**							
Step (1) *	-149.824	0.000	0.000	-149.824	0.80	0	1.72
Step (2) O_2*	-160.801	0.169	0.097	-160.729	0	0	0.808
Step(3)(O+OH)*	-168.774	0.511	0.087	-168.350	0.80	0	-2.568
Step(4) O*	-158.734	0.117	0.020	-158.636	0.80	0	-2.956
Step(5) OH*	-160.844	0.430	0.030	-160.445	0.80	0	-0.519
**U= 1.23 V**							
Step (1) *	-149.824	0.000	0.000	-149.824	1.23	0	0
Step (2) O_2*	-160.801	0.169	0.097	-160.729	0	0	-0.912
Step(3)(O+OH)*	-168.774	0.511	0.087	-168.350	1.23	0	-3.858
Step(4) O*	-158.734	0.117	0.020	-158.636	1.23	0	-3.816
Step(5) OH*	-160.844	0.430	0.030	-160.445	1.23	0	-0.949
**g-P-N1-Pt1**							
**U=0.00 V**							
Step (1) *	-155.694	0	0	-155.694	0	0	4.92
Step(2) O_2*	-167.483	0.132	0.155	-167.506	0	0	3.101
Step(3) OOH*	-171.618	0.449	0.185	-171.354	0	0	2.698
Step(4) O*	-162.753	0.071	0.069	-162.751	0	0	0.400
Step(5) OH*	-167.311	0.341	0.137	-167.108	0	0	-0.512
**U=0.20 V**							

**Step (1) ***	**-155.694**	**0**	**0**	**-155.694**	**0.20**	**0**	**4.12**
**Step(2) O_2***	**-167.483**	**0.132**	**0.155**	**-167.506**	**0**	**0**	**2.301**
**Step(3) OOH***	**-171.618**	**0.449**	**0.185**	**-171.354**	**0.20**	**0**	**2.098**
**Step(4) O***	**-162.753**	**0.071**	**0.069**	**-162.751**	**0.20**	**0**	**0.000**
**Step(5) OH***	**-167.311**	**0.341**	**0.137**	**-167.108**	**0.20**	**0**	**-0.712**
**U=0.40 V**							
**Step (1) ***	**-155.694**	**0**	**0**	**-155.694**	**0.40**	**0**	**3.32**
**Step(2) O_2***	**-167.483**	**0.132**	**0.155**	**-167.506**	**0**	**0**	**1.501**
**Step(3) OOH***	**-171.618**	**0.449**	**0.185**	**-171.354**	**0.40**	**0**	**1.498**
**Step(4) O***	**-162.753**	**0.071**	**0.069**	**-162.751**	**0.40**	**0**	**-0.400**
**Step(5) OH***	**-167.311**	**0.341**	**0.137**	**-167.108**	**0.40**	**0**	**-0.912**
**U= 0.80 V**							
**Step (1) ***	**-155.694**	**0**	**0**	**-155.694**	**0.80**	**0**	**1.72**
**Step(2) O_2***	**-167.483**	**0.132**	**0.155**	**-167.506**	**0**	**0**	**-0.099**
**Step(3) OOH***	**-171.618**	**0.449**	**0.185**	**-171.354**	**0.80**	**0**	**0.298**
**Step(4) O***	**-162.753**	**0.071**	**0.069**	**-162.751**	**0.80**	**0**	**-1.200**
**Step(5) OH***	**-167.311**	**0.341**	**0.137**	**-167.108**	**0.80**	**0**	**-1.312**
**U= 1.23 V**							
**Step (1) ***	**-155.694**	**0**	**0**	**-155.694**	**1.23**	**0**	**0**
**Step(2) O_2***	**-167.483**	**0.132**	**0.155**	**-167.506**	**0**	**0**	**-1.819**
**Step(3) OOH***	**-171.618**	**0.449**	**0.185**	**-171.354**	**1.23**	**0**	**-0.992**
**Step(4) O***	**-162.753**	**0.071**	**0.069**	**-162.751**	**1.23**	**0**	**-2.060**
**Step(5) OH***	**-167.311**	**0.341**	**0.137**	**-167.108**	**1.23**	**0**	**-1.742**
**Pt(111)**							
**U=0.00 V**							
**Step (1) ***	**-152.734**	**0**	**0**	**-152.734**	**0**	**0**	**4.92**
**Step(2) O_2***	**-163.302**	**0.144**	**0.121**	**-163.280**	**0**	**0**	**4.366**
**Step(3) OOH***	**-167.146**	**0.432**	**0.198**	**-166.911**	**0**	**0**	**4.180**
**Step(4) O***	**-158.452**	**0.063**	**0.049**	**-158.437**	**0**	**0**	**1.752**
**Step(5) OH***	**-162.827**	**0.363**	**0.072**	**-162.536**	**0**	**0**	**1.099**
**U=0.20 V**							
**Step (1) ***	**-152.734**	**0**	**0**	**-152.734**	**0.20**	**0**	**4.12**
**Step(2) O_2***	**-163.302**	**0.144**	**0.121**	**-163.280**	**0**	**0**	**3.566**
**Step(3) OOH***	**-167.146**	**0.432**	**0.198**	**-166.911**	**0.20**	**0**	**3.580**
**Step(4) O***	**-158.452**	**0.063**	**0.049**	**-158.437**	**0.20**	**0**	**1.352**
**Step(5) OH***	**-162.827**	**0.363**	**0.072**	**-162.536**	**0.20**	**0**	**0.899**
**U=0.40 V**							
**Step (1) ***	**-152.734**	**0**	**0**	**-152.734**	**0.40**	**0**	**3.32**
**Step(2) O_2***	**-163.302**	**0.144**	**0.121**	**-163.280**	**0**	**0**	**2.766**
**Step(3) OOH***	**-167.146**	**0.432**	**0.198**	**-166.911**	**0.40**	**0**	**2.980**
**Step(4) O***	**-158.452**	**0.063**	**0.049**	**-158.437**	**0.40**	**0**	**0.952**
**Step(5) OH***	**-162.827**	**0.363**	**0.072**	**-162.536**	**0.40**	**0**	**0.699**
**U= 0.80 V**							

**Step (1) ***	**-152.734**	**0**	**0**	**-152.734**	**0.80**	**0**	**1.72**
**Step(2) O_2***	**-163.302**	**0.144**	**0.121**	**-163.280**	**0**	**0**	**1.166**
**Step(3) OOH***	**-167.146**	**0.432**	**0.198**	**-166.911**	**0.80**	**0**	**1.780**
**Step(4) O***	**-158.452**	**0.063**	**0.049**	**-158.437**	**0.80**	**0**	**0.152**
**Step(5) OH***	**-162.827**	**0.363**	**0.072**	**-162.536**	**0.80**	**0**	**0.299**
**U= 1.23 V**							
**Step (6) ***	**-152.734**	**0**	**0**	**-152.734**	**1.23**	**0**	**0**
**Step(2) O_2***	**-163.302**	**0.144**	**0.121**	**-163.280**	**0**	**0**	**-0.554**
**Step(3) OOH***	**-167.146**	**0.432**	**0.198**	**-166.911**	**1.23**	**0**	**0.490**
**Step(4) O***	**-158.452**	**0.063**	**0.049**	**-158.437**	**1.23**	**0**	**-0.708**
**Step(5) OH***	**-162.827**	**0.363**	**0.072**	**-162.536**	**1.23**	**0**	**-0.131**

Supplementary Table 8. DFT total energies (E), zero-point energies (ZPE), entropies multiplied by T (= 298.15 K) (TS), free energies (G) and relative free energies (ΔG) of ORR intermediates in alkaline medium.

	**E(eV)**	**ZPE(eV)**	**TS(eV)**	**G(eV)**	**ΔG_U (eV)**	**ΔG_{pH}(eV)**	**ΔG(eV)**
**g-P-N1**							
**U=0.40 V**							
**Step(2) O_2***	**-160.801**	**0.169**	**0.097**	**-160.729**	**0**	**0**	**-0.912**
**Step(3)(O+OH)***	**-168.774**	**0.511**	**0.087**	**-168.350**	**0.40**	**0.769**	**-3.918**
**Step(4) O***	**-158.734**	**0.117**	**0.020**	**-158.636**	**0.40**	**0.769**	**-3.938**
**Step(5) OH***	**-160.844**	**0.430**	**0.030**	**-160.445**	**0.40**	**0.769**	**-1.131**
**Step (6) ***	**-149.824**	**0.000**	**0.000**	**-149.824**	**0.40**	**0.769**	**-0.243**
**U=0.20 V**							
**Step (2) O_2***	**-160.801**	**0.169**	**0.097**	**-160.729**	**0**	**0**	**-0.912**
**Step(3)(O+OH)***	**-168.774**	**0.511**	**0.087**	**-168.350**	**0.20**	**0.769**	**-4.118**
**Step(4) O***	**-158.734**	**0.117**	**0.020**	**-158.636**	**0.20**	**0.769**	**-4.338**
**Step(5) OH***	**-160.844**	**0.430**	**0.030**	**-160.445**	**0.20**	**0.769**	**-1.731**
**Step (6) ***	**-149.824**	**0.000**	**0.000**	**-149.824**	**0.20**	**0.769**	**-1.043**
**U=0.00 V**							
**Step (2) O_2***	**-160.801**	**0.169**	**0.097**	**-160.729**	**0**	**0**	**-0.912**
**Step(3)(O+OH)***	**-168.774**	**0.511**	**0.087**	**-168.350**	**0.00**	**0.769**	**-4.318**
**Step(4) O***	**-158.734**	**0.117**	**0.020**	**-158.636**	**0.00**	**0.769**	**-4.738**
**Step(5) OH***	**-160.844**	**0.430**	**0.030**	**-160.445**	**0.00**	**0.769**	**-2.331**
**Step (6) ***	**-149.824**	**0.000**	**0.000**	**-149.824**	**0.00**	**0.769**	**-1.843**
**U= -0.40 V**							
**Step (2) O_2***	**-160.801**	**0.169**	**0.097**	**-160.729**	**0**	**0**	**-0.912**
**Step(3)(O+OH)***	**-168.774**	**0.511**	**0.087**	**-168.350**	**-0.40**	**0.769**	**-4.718**
**Step(4) O***	**-158.734**	**0.117**	**0.020**	**-158.636**	**-0.40**	**0.769**	**-5.538**
**Step(5) OH***	**-160.844**	**0.430**	**0.030**	**-160.445**	**-0.40**	**0.769**	**-3.531**
**Step (6) ***	**-149.824**	**0.000**	**0.000**	**-149.824**	**-0.40**	**0.769**	**-3.443**
**U= -0.83 V**							
**Step (2) O_2***	**-160.801**	**0.169**	**0.097**	**-160.729**	**0**	**0**	**-0.912**

Step(3)(O+OH)*	-168.774	0.511	0.087	-168.350	-0.83	0.769	-5.148
Step(4) O*	-158.734	0.117	0.020	-158.636	-0.83	0.769	-6.398
Step(5) OH*	-160.844	0.430	0.030	-160.445	-0.83	0.769	-4.821
Step (6) *	-149.824	0.000	0.000	-149.824	-0.83	0.769	-5.163
g-P-N1-Pt1							
U=0.40 V							
Step(2) O_2*	-167.483	0.132	0.155	-167.506	0	0	-1.819
Step(3) OOH*	-171.618	0.449	0.185	-171.354	0.40	0.769	-1.053
Step(4) O*	-162.753	0.071	0.069	-162.751	0.40	0.769	-2.182
Step(5) OH*	-167.311	0.341	0.137	-167.108	0.40	0.769	-1.924
Step (6) *	-155.694	0	0	-155.694	0.40	0.769	-0.243
U=0.20 V							
Step(2) O_2*	-167.483	0.132	0.155	-167.506	0	0	-1.819
Step(3) OOH*	-171.618	0.449	0.185	-171.354	0.20	0.769	-1.253
Step(4) O*	-162.753	0.071	0.069	-162.751	0.20	0.769	-2.582
Step(5) OH*	-167.311	0.341	0.137	-167.108	0.20	0.769	-2.524
Step (6) *	-155.694	0	0	-155.694	0.20	0.769	-1.043
U=0.00 V							
Step(2) O_2*	-167.483	0.132	0.155	-167.506	0	0	-1.819
Step(3) OOH*	-171.618	0.449	0.185	-171.354	0.00	0.769	-1.453
Step(4) O*	-162.753	0.071	0.069	-162.751	0.00	0.769	-2.982
Step(5) OH*	-167.311	0.341	0.137	-167.108	0.00	0.769	-3.124
Step (6) *	-155.694	0	0	-155.694	0.00	0.769	-1.843
U= -0.40 V							
Step(2) O_2*	-167.483	0.132	0.155	-167.506	0	0	-1.819
Step(3) OOH*	-171.618	0.449	0.185	-171.354	-0.40	0.769	-1.853
Step(4) O*	-162.753	0.071	0.069	-162.751	-0.40	0.769	-3.782
Step(5) OH*	-167.311	0.341	0.137	-167.108	-0.40	0.769	-4.324
Step (6) *	-155.694	0	0	-155.694	-0.40	0.769	-3.443
U= -0.83 V							
Step(2) O_2*	-167.483	0.132	0.155	-167.506	0	0	-1.819
Step(3) OOH*	-171.618	0.449	0.185	-171.354	-0.83	0.769	-2.283
Step(4) O*	-162.753	0.071	0.069	-162.751	-0.83	0.769	-4.642
Step(5) OH*	-167.311	0.341	0.137	-167.108	-0.83	0.769	-5.614
Step (6) *	-155.694	0	0	-155.694	-0.83	0.769	-5.163
Pt(111)							
U=0.40 V							
Step(2) O_2*	-163.302	0.144	0.121	-163.280	0	0	-0.554
Step(3) OOH*	-167.146	0.432	0.198	-166.911	0.40	0.769	0.429
Step(4) O*	-158.452	0.063	0.049	-158.437	0.40	0.769	-0.829
Step(5) OH*	-162.827	0.363	0.072	-162.536	0.40	0.769	-0.313
Step (6) *	-152.734	0	0	-152.734	0.40	0.769	-0.243
U=0.20 V							

**Step(2) O_2***	**-163.302**	**0.144**	**0.121**	**-163.280**	**0**	**0**	**-0.554**
**Step(3) OOH***	**-167.146**	**0.432**	**0.198**	**-166.911**	**0.20**	**0.769**	**0.229**
**Step(4) O***	**-158.452**	**0.063**	**0.049**	**-158.437**	**0.20**	**0.769**	**-1.229**
**Step(5) OH***	**-162.827**	**0.363**	**0.072**	**-162.536**	**0.20**	**0.769**	**-0.913**
**Step (6) ***	**-152.734**	**0**	**0**	**-152.734**	**0.20**	**0.769**	**-1.043**
**U=0.00 V**							
**Step(2) O_2***	**-163.302**	**0.144**	**0.121**	**-163.280**	**0**	**0**	**-0.554**
**Step(3) OOH***	**-167.146**	**0.432**	**0.198**	**-166.911**	**0.00**	**0.769**	**0.029**
**Step(4) O***	**-158.452**	**0.063**	**0.049**	**-158.437**	**0.00**	**0.769**	**-1.629**
**Step(5) OH***	**-162.827**	**0.363**	**0.072**	**-162.536**	**0.00**	**0.769**	**-1.513**
**Step (6) ***	**-152.734**	**0**	**0**	**-152.734**	**0.00**	**0.769**	**-1.843**
**U= -0.40 V**							
**Step(2) O_2***	**-163.302**	**0.144**	**0.121**	**-163.280**	**0**	**0**	**-0.554**
**Step(3) OOH***	**-167.146**	**0.432**	**0.198**	**-166.911**	**-0.40**	**0.769**	**-0.371**
**Step(4) O***	**-158.452**	**0.063**	**0.049**	**-158.437**	**-0.40**	**0.769**	**-2.429**
**Step(5) OH***	**-162.827**	**0.363**	**0.072**	**-162.536**	**-0.40**	**0.769**	**-2.713**
**Step (6) ***	**-152.734**	**0**	**0**	**-152.734**	**-0.40**	**0.769**	**-3.443**
**U= -0.83 V**							
**Step(2) O_2***	**-163.302**	**0.144**	**0.121**	**-163.280**	**0**	**0**	**-0.554**
**Step(3) OOH***	**-167.146**	**0.432**	**0.198**	**-166.911**	**-0.83**	**0.769**	**-0.801**
**Step(4) O***	**-158.452**	**0.063**	**0.049**	**-158.437**	**-0.83**	**0.769**	**-3.289**
**Step(5) OH***	**-162.827**	**0.363**	**0.072**	**-162.536**	**-0.83**	**0.769**	**-4.003**
**Step (6) ***	**-152.734**	**0**	**0**	**-152.734**	**-0.83**	**0.769**	**-5.163**

Supplementary References

1. Ravel, B., Newville, M., Athena, Artemis, Hephaestus: data analysis for X-ray absorption spectroscopy using IFEFFIT. *J. Synch. Radi.* **12**, 537-541(2005).

2. Deleon, J. M., Rehr, J. J., Zabinsky, S. I., Albers, R. C., Abinitio Curved-Wave X-Ray-Absorption Fine-Structure. *Phys. Rev. B* **44**, 4146-4156(1991).

3. Rehr, J. J., Deleon, J. M., Zabinsky, S. I., Albers, R. C., Theoretical X-Ray Absorption Fine-Structure Standards. *J. Am. Chem. Soc.* **113**, 5135-5140(1991).

4. Zabinsky, S. I., Rehr, J. J., Ankudinov, A., Albers, R. C., Eller, M. J., Multiple-Scattering Calculations of X-Ray-Absorption Spectra. *Phys, Rev, B* **52**,2995-3009(1995).

5. Xiao, M., Zhu, J., Feng, L., Liu, C., Xing, W. Meso/Macroporous Nitrogen-Doped Carbon Architectures with Iron Carbide Encapsulated in Graphitic Layers as an Efficient and Robust Catalyst for the Oxygen Reduction Reaction in Both Acidic and Alkaline Solutions. *Adv. Mater.***27**, 2521–2527(2015).

6. Hu, Y., Jensen, J. O., Zhang, W. Cleemann, La. N. Xing, W., et al. Hollow Spheres of Iron Carbide Nanoparticles Encased in Graphitic Layers as Oxygen Reduction Catalysts. *Angew. Chem. Int. Ed.* **53**, 3675 –3679(2014).

7. Lin, L., Zhu, Q., Xu, A. W. Noble-Metal-Free Fe–N/C Catalyst for Highly Efficient Oxygen Reduction Reaction under Both Alkaline and Acidic Conditions. *J. Am. Chem. Soc.* **136**, 11027−11033(2014).

8. Liu, L., Yang, X. F., Ma, N. Liu, H. T., Xia, Y. Z., Chen, C. M., Yang, D. J., Yao, X. D. Scalable and Cost-Effective Synthesis of Highly Efficient Fe2N-Based Oxygen Reduction Catalyst Derived from Seaweed Biomass. *Small* **12**, 1295-1301(2016).

9. Wu, Z. Y., Xu, X. X., Hu, B. C., Liang, H. W., Lin, Y., Chen, L. F., Yu, S. H. Iron Carbide Nanoparticles Encapsulated in Mesoporous Fe-N-Doped Carbon Nanofibers for Efficient Electrocatalysis. *Angew. Chem. Int. Ed.* **54**, 8179–8183(2015).
10. Shui, J., Chen, C., Grabstanowicz, L., Zhao, D., Liu, D. J. Highly efficient nonprecious metal catalyst prepared with metal–organic framework in a continuous carbon nanofibrous network. *PNAS* **112**, 10629–10634(2015).
11. Wu, G., More, K. L., Johnston, C. M., Zelenay, P. High-Performance Electrocatalysts for Oxygen Reduction Derived from Polyaniline, Iron, and Cobalt. *Science* **332**, 443–447(2011)
12. Wu, G. P., Wang, J., Ding, W., *et al*. A Strategy to Promote the Electrocatalytic Activity of Spinels for Oxygen Reduction by Structure Reversal. *Angew. Chem. Int. Ed*. **55**, 1340 –1344(2016).
13. Shang, L., Yu, H. J., Huang, X. *et al*. Well-Dispersed ZIF-Derived Co,N-Co-doped Carbon Nanoframes through Mesoporous-Silica-Protected Calcination as Efficient Oxygen Reduction Electrocatalysts. *Adv. Mater. 28*, 1668–1674(2016).
14. Jiang, K., Wang, P., Guo, S., Zhang, X., Shen, X., Lu, G., Su, D., Huang, X. Ordered PdCu-Based Nanoparticles as Bifunctional Oxygen-Reduction and Ethanol-Oxidation Electrocatalysts. *Angew.Chem.Int.Ed.* **55**,9030-9035(2016).
15. Zhang, J., Zhao, Z., Xia, Z., Dai, L. A metal-free bifunctional electrocatalyst for oxygen reduction and oxygen evolution reactions. *Nature Nanotech.* **10**,444–452(2015).
16. Niu, W., Li, L., *et al*. Mesoporous N-Doped Carbons Prepared with Thermally Removable Nanoparticle Templates: An Efficient Electrocatalyst for Oxygen Reduction Reaction. *J. Am. Chem.Soc.* **137**, 5555–5562(2015).
17. Chung, H. T., Won, J. H., Zelenay, P. Active and stable carbon nanotube/nanoparticle composite electrocatalyst for oxygen reduction. *Nat. Commun.*,**4**,1922/1-5(2013).
18. Gong, K., Du, F., Xia, Z., Durstock, M., Dai, L. Nitrogen-Doped Carbon Nanotube Arrays with High Electrocatalytic Activity for Oxygen Reduction, *Science* **323**, 760–764(2009).
19. Yin, P., Yao, T. *et al*., Single Cobalt Atoms with Precise N-Coordination as Superior Oxygen Reduction Reaction Catalysts. *Angew. Chem. Int. Ed*., **55**, 10800-10805(2016)
20. Proietti, E., Jaouen, F., Lefèvre, M., *et al*. Iron-based cathode catalyst with enhanced power density in polymer electrolyte membrane fuel cells. Nat. Commun. **2**, 416/1-416/9(2011).
21. Wang, Y.-C., Lai, Y.-J., *et al*. S-Doping of an Fe/N/C ORR Catalyst for Polymer Electrolyte Membrane Fuel Cells with High Power Density. Angew. Chem. Int. Ed. **54**, 9907-9910(2015).
22. Lefevre, M., Proietti, E., Jaouen, F., Dodelet, J. P. Iron-Based Catalysts with Improved Oxygen Reduction Activity in Polymer Electrolyte Fuel Cells. *Science* **324**, 71-74(2009).
23. Kresse, G., Hafner, J. Ab initio molecular dynamics for liquid metals. *Phys. Rev. B* **47**,558-561(1993).
24. Kresse, G., Hafner, J. Ab initio molecular-dynamics simulation of the liquid-metal–amorphous-semiconductor transition in germanium. *Phys. Rev. B* **49**, 14251-14269(1994).
25. Kresse, G., Furthmüller, J. Efficient iterative schemes for ab initio total-energy calculations using a plane-wave basis set. *Phys. Rev. B* **54**, 11169-11186(1996).
26. Kresse, G., Furthmüller, J. Efficiency of ab-initio total energy calculations for metals and semiconductors using a plane-wave basis set. *Comp. Mate.r Sci.* **6**, 15-50(1996).
27. Blöchl, P. E. Projector augmented-wave method. *Phy.s Rev. B* **50**,17953-17979(1994).
28. Kresse, G., Joubert, D. From ultrasoft pseudopotentials to the projector augmented-wave method. *Phys. Rev. B* **59**, 1758-1775(1999).
29. Perdew, J. P., Burke, K., Ernzerhof, M. Generalized Gradient Approximation Made Simple. *Phys. Rev. Lett.*

77, 3865-3868(1996).

30. Monkhorst, H. J., Pack, J. D. Special points for Brillouin-zone integrations. *Phys. Rev. B* **13**, 5188-5192(1976).

31. Nørskov, J. K., *et al.* Origin of the Overpotential for Oxygen Reduction at a Fuel-Cell Cathode. *J. Phys. Chem. B* **108**,17886-17892(2004).

32. Yu, L., Pan, X., Cao, X., Hu, P., Bao, X. Oxygen reduction reaction mechanism on nitrogen-doped graphene: A density functional theory study. *J Catal.* **282**,183-190(2011).

33. Kattel, S., Atanassov, P., Kiefer, B. Density Functional Theory Study of Ni–N_x/C Electrocatalyst for Oxygen Reduction in Alkaline and Acidic Media. *J. Phys. Chem. C* **116**, 17378-17383(2012).

34. Srinivasu, K., Ghosh, S. K. Transition Metal Decorated Graphyne: An Efficient Catalyst for Oxygen Reduction Reaction. *J. Phys. Chem. C* **117**,26021-26028(2013).

35. Lauritsen, J. V., Besenbacher, F. New insight into nanocatalysis from atom-resolved scanning tunneling microscopy. *Nova. Acta. Leopoldina* **92**, 21-28 (2005).

36. Mawhinney, D. B., *et al.* Surface defect site density on single walled carbon nanotubes by titration. *Chem. Phy.s Lett.* **324**,213-216(2000).
